# Modified FOLFIRINOX versus gemcitabine plus oxaliplatin as first-line chemotherapy for patients with locally advanced or metastatic cholangiocarcinoma: a retrospective comparative study

**DOI:** 10.1186/s12885-021-08549-2

**Published:** 2021-07-16

**Authors:** Lu Zou, Xuechuan Li, Xiangsong Wu, Jiujie Cui, Xuya Cui, Xiaoling Song, Tai Ren, Xusheng Han, Yidi Zhu, Huaifeng Li, Wenguang Wu, Xu’an Wang, Wei Gong, Liwei Wang, Maolan Li, Wan Yee Lau, Yingbin Liu

**Affiliations:** 1 Department of General Surgery, Xinhua Hospital Affiliated to Shanghai Jiao Tong University School of Medicine, Shanghai, 200092 China; 2 Department of Biliary-Pancreatic Surgery, Renji Hospital, School of Medicine, Shanghai Jiao Tong University, Shanghai, 200127 China; 3 Shanghai Key Laboratory of Biliary Tract Disease Research, Shanghai, 200092 China; 4 Shanghai Cancer Institute, Renji Hospital, Shanghai Jiao Tong University School of Medicine, Shanghai, 200032 China; 5 Department of Medical Oncology, Shanghai Cancer Institute, Renji Hospital, School of Medicine, Shanghai, 200127 China; 6 Faculty of Medicine, The Chinese University of Hong Kong, Shatin, Hong Kong SAR, China

**Keywords:** mFOLFIRINOX chemotherapy gemcitabine cholangiocarcinoma

## Abstract

**Background:**

Gemcitabine plus platinum as the first-line chemotherapy for cholangiocarcinoma (CCA) has limited efficacy. The aim of this study was to evaluate the effectiveness of modified FOLFIRINOX (mFOLFIRINOX) compared to that of gemcitabine plus oxaliplatin (Gemox) for patients with locally advanced or metastatic CCA.

**Methods:**

From January 2016 to December 2019, consecutive patients who were diagnosed with locally advanced or metastatic CCA were treated with either mFOLFIRINOX or Gemox as a first-line chemotherapy. The main endpoint was Progression free survival (PFS). The second endpoints were Overall survival (OS), Disease control rate (DCR) and incidence of severe toxicity (grade 3–4). Tumors were evaluated at baseline and thence every 4–6 weeks. The study was designed and carried out in accordance with the principles of the declaration of Helsinki, approved by the Ethics Committee of Xinhua Hospital Affiliated to Shanghai Jiaotong University School of Medicine (XHEC-D-2020-154) and registered with ClinicalTrials.gov, number NCT04305288 (registration date: 12/03/2020).

**Results:**

Of 49 patients in this study, 27 were in the FOLFIRINOX regimen group and 22 in the Gemox regimen group. There were no significant differences between groups in baseline characteristics. The DCR was 77.8% in the mFOLFIRINOX group and 63.5% in the Gemox group. The corresponding median PFS was 9.9 months (95% confidence interval [CI], 7.3–12.4) in the mFOLFIRINOX group versus 6.4 months (95% CI,3.6–9.2, *p* = 0.040) in the Gemox group. The corresponding median OS was 15.7 months (95% CI, 12.5–19.0) versus 12.0 months (95% CI, 9.3–14.8, *p* = 0.099). Significantly more grade 3–4 vomiting occurred in the mFOLFIRINOX than the Gemox groups (7 (25.9%) vs 1 (4.5%), *p* = 0.044).

**Conclusions:**

First-line mFOLFIRINOX offered more promising results in patients with advanced or metastatic CCA.

## Introduction

Cholangiocarcinoma (CCA), which includes intrahepatic, hilar and distal CCA, is a heterogeneous group of rare tumors [1, 2]. Most patients were diagnosed at advanced stage and missed the opportunity for R0 surgical resection. The prognosis for advanced CCA is less favorable with a median survival of less than 12 months and an overall survival rate (OS) of 5 years of approximately 5% [3]. Gemcitabine plus platinum, as the first-line chemotherapy for CCA, has limited efficacy (OS: 11.7 months) [4]. Gemcitabine plus oxaliplatin (Gemox) has similar efficacy compared with gemcitabine plus cisplatin (weighted median OS: oxaliplatin group vs cisplatin; 9.5 months vs 9.7 months) [5]. The progression-free survival (PFS) after second-line therapy is only about 3 months [6]. Targeted therapy and immunotherapy have the potential to become an option in the treatment of CCA [7]. However, more studies are needed to confirm the efficacy of these molecule drugs. The ESMO clinical guidelines recommend cisplatin/gemcitabine chemotherapy regimen or participation in clinical trials for patients with locally advanced or metastatic CCA [8].

Modified FOLFIRINOX (mFOLFIRINOX) regimen (irinotecan, fluorouracil, leucovorin and oxaliplatin) resulted in a longer OS than gemcitabine alone [9] (11.1 vs 6.8 months), and has become the first-line chemotherapy of metastatic pancreatic cancer. As therapeutic similarities in sensitivity to fluorouracil, platinum and gemcitabine exist between CCA and pancreatic cancer [10], mFOLFIRINOX might show better efficacy than Gemox in treating patients with CCA. Ulusakarya et al [11] had reported that the median OS of patients with advanced biliary tract cancer treated with first-line FOLFIRINOX was as long as 15 months. Recently, Angela et al [12] reported mFOLFOX (folinic acid, fluorouracil, and oxaliplatin) improved the prognosis of patients with advanced biliary tract cancer after progression on cisplatin and gemcitabine. These studies suggested that FOLFIRINOX might be a potential treatment option of CCA. This study was conducted to evaluate the effectiveness and safety of mFOLFIRINOX compared to Gemox for patients with locally advanced or metastatic CCA.

## Methods

### Patients

This is a retrospective study on consecutive patients with intrahepatic, hilar, or distal CCA at locally advanced (non-resectable) or metastatic stage who were treated with either mFOLFIRINOX or Gemox as a first-line therapy from January 2016 to December 2019 at Xinhua Hospital Affiliated to Shanghai Jiaotong University School of Medicine. All patients were diagnosed with treatment-naive CCA. Metastasis was defined as distant metastasis on medical imaging. Locally advanced disease was defined as inability to undergo radical resection in the absence of distant metastasis after assessment by an experienced surgeon. The study was censored on September 30, 2020. All patients had signed informed consent forms. The study was designed and carried out in accordance with the principles of the declaration of Helsinki, approved by the Ethics Committee of Xinhua Hospital Affiliated to Shanghai Jiaotong University School of Medicine (XHEC-D-2020-154) and registered with ClinicalTrials.gov, number NCT04305288 (registration date: 12/03/2020).

### Treatment and assessment

The mFOLFIRINOX regimen consisted of irinotecan 150 mg/m^2^, oxaliplatin 65 mg/m^2^, Calcium folinate 400 mg/m^2^, fluorouracil 400 mg/m^2^ and continuous fluorouracil 2400 mg/m^2^ (46 h), in a 2-week schedule. The Gemox regimen consisted of 100 mg/m^2^ oxaliplatin followed by 1000 mg/m^2^ gemcitabine on days 1 and 8 once every 3 weeks. The main endpoint was Progression free survival (PFS). The second endpoints were Overall survival (OS), Disease control rate (DCR) and incidence of severe toxicity (grade 3–4). Tumors were evaluated at baseline and thence every 4–6 weeks during treatment using magnetic resonance imaging or computed tomography. Response and progression were assessed using the Response Evaluation Criteria in Solid Tumors (RECIST) version 1.1.

Patients underwent a complete biological examination before each treatment cycle, including full blood count and biochemistry of liver and kidney. Safety was evaluated using the Common Terminology Criteria for Adverse Events (version 4.0). The rates of grade 3–4 adverse events between groups were compared.

### Statistical analysis

The Wilcoxon rank was used to compare continuous data with skewed distributions. Categorical variables were compared using the chi-square test or Fisher’s exact test. PFS was defined as the time from diagnosis to disease progression or death, whichever occurred first. OS was defined as the time from diagnosis to death from any cause. PFS and OS were analyzed using the Kaplan–Meier method and the log-rank test. Univariable and multivariable analyses were performed using the Cox proportional hazards regression model to determine prognostic factors for PFS and OS. The variables that showed potential associations with OS or PFS in univariable analysis (*p* < 0.2) were further tested in multivariable analyses. All analyses were performed using SPSS version 22.0 (IBM Corp, Armonk, NY, USA). A two-sided *p*-value of < 0.05 was considered statistically significant.

## Results

### Patient characteristics

From January 2016 to December 2019, 49 patients were included in the study. Table 1 summarized the baseline characteristics of these patients. Twenty-seven patients were treated with mFOLFIRINOX (15 males and 12 females, median age 58 years), and 22 patients with Gemox (8 males and 14 females, median age 56.5 years). All patients had a good general condition (ECOG score of 0 or 1). Most patients were diagnosed with metastatic CCA (mFOLFIRINOX vs Gemox: 21 (77.8%) in 27 vs 14 (63.6%) in 22). Liver metastasis was common in both groups. There was no significant difference in baseline characteristics among groups.

**Table 1:** Baseline patient characteristics

Characteristic	mFOLFIRINOX (***n*** = 27)	Gemox (***n*** = 22)	***P*** value
**Age**			
Median, range	58 (41–73)	56.5 (36–75)	0.817
**Sex**			0.181
Male	15 (55.6)	8 (36.4)	
Female	12 (44.4)	14 (63.6)	
**ECOG**			0.407
0	13 (48.1)	8 (36.4)	
1	14 (51.9)	14 (63.6)	
**Primary tumor sites**			0.434
Intrahepatic	16 (59.3)	9 (40.9)	
Hilar	9 (33.3)	11 (50.0)	
Distal	2 (7.4)	2 (9.1)	
**Disease status**			0.276
Locally advanced	6 (22.2)	8 (36.4)	
Metastatic	21 (77.8)	14 (63.6)	
**Metastatic site**			
Liver	17 (63.0)	13 (59.1)	0.782
Lung	5 (18.5)	2 (9.1)	–
Bone	1 (3.7)	0 (0)	–
Other	1 (3.7)	1 (4.5)	–
**CA19–9**			
Median, range	172.0 (6.5–18,940)	151.3 (1.7–26,666)	0.680

ECOG Eastern Cooperative Oncology Group

Data are presented as n (%) for categorical variables and as median (range) for continuous variables

### Efficacy

The median treatment was 14 cycles for mFOLFIRINOX and 8 cycles for Gemox. Partial response (PR) occurred in 9(33.3%) patients in the mFOLFIRINOX regimen and 5(22.7%) patients in the Gemox regimen (Table 2). No patients achieved complete response (CR). The disease control rates (DCR) were 77.8 and 63.5% in the mFOLFIRINOX regimen and Gemox regimen, respectively.

**Table 2:** Best tumor response according to the Response Evaluation Criteria in Solid Tumors (RECIST) 1.1

	mFOLFIRINOX (***n*** = 27) N (%)	Gemox (***n*** = 22) N (%)	***p*** value
**Complete response**	0 (0)	0 (0)	–
**Partial response**	9 (33.3)	5 (22.7)	–
**Stable disease**	12 (44.4)	9 (40.9)	–
**Progressive disease**	6 (22.2)	8 (36.4)	–
**Objective response rate**	9 (33.3)	5 (22.7)	0.530
**Disease control rate**	21 (77.8)	14 (63.5)	0.276

**Fig. 1:**
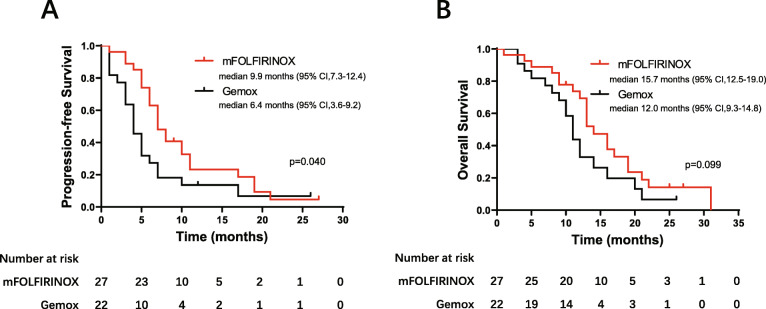
Progression-free survival (**A**) and overall survival (**B**) in patients receiving mFOLFIRINOX (*n* = 27) and Gemox (*n* = 22)

The Kaplan–Meier curves for PFS and OS were shown in Fig. 1. Median PFS was 9.9 months (95% CI 7.3–12.4) for the mFOLFIRINOX group versus 6.4 months (95% CI 3.6–9.2) for the Gemox group (*p* = 0.040). The corresponding median OS was 15.7 months (95% CI 12.5–19.0) versus 12.0 months (95% CI 9.3–14.8), respectively (*p* = 0.099).

Multivariable analyses are shown in Tables 3 and 4. For PFS, use of mFOLFIRINOX versus Gemox (HR = 0.353 [95% CI, 0.180–0.694]; *p* = 0.003), hilar CCA versus intrahepatic CCA (HR = 2.149 [95% CI, 1.113–4.151]; *p* = 0.023), presence of liver metastasis (HR = 3.096 [95% CI, 1.535–6.246]; *p* = 0.002) and a high level of CA19–9 (HR = 3.622 [95% CI, 1.540–8.523]; *p* = 0.003) were independent prognostic factors. For OS, a high ECOG level (HR = 2.148 [95% CI, 1.028–4.488]; *p* = 0.042), hilar CCA versus intrahepatic CCA (HR = 2.123 [95% CI, 1.023–4.402]; *p* = 0.043) and a high level of CA19–9 (HR = 4.972 [95% CI, 1.768–13.980]; *p* = 0.002) were independent prognostic factors.

**Table 3:** Univariable and multivariable analysis for progression-free survival (PFS)

Variables	Univariable analysis		Multivariable analysis	
	HR (95% CI)	*P* value	HR (95% CI)	*P* value
**Treatment regimen**				
mFOLFIRINOX vs Gemox	0.559 (0.307–1.020)	**0.040**	0.353 (0.180–0.694)	**0.003**
**Age**				
>58 vs ≤58	1.060 (0.579–1.939)	0.851		
**Sex**				
male vs female	0.844 (0.626–1.139)	0.268		
**ECOG**				
1 vs 0	1.220 (0.664–2.242)	0.523		
**Primary tumor sites**				
Intrahepatic	Ref		Ref	
Hilar	1.599 (0.869–2.944)	0.131	2.149 (1.113–4.151)	**0.023**
Distal	0.512 (0.120–2.191)	0.367	0.493 (0.111–2.185)	0.352
**Disease status**				
Metastasis vs Locally advanced	1.111 (0.790–1.564)	0.545		
**Liver metastasis**	1.658 (0.891–3.088)	0.111	3.096 (1.535–6.246)	**0.002**
**CA19–9**				
>40 vs ≤40	1.867 (0.863–4.036)	0.113	3.622 (1.540–8.523)	**0.003**

Bold values are statistically significant (*p* ≤ 0.05)

*Ref* reference, *HR* hazard ratio, *CI* confidence interval, *ECOG* Eastern Cooperative Oncology Group

**Table 4:** Univariable and multivariable analysis for overall survival (OS)

Variables	Univariable analysis		Multivariable analysis	
	HR (95% CI)	*P* value	HR (95% CI)	*P* value
**Treatment regimen**				
mFOLFIRINOX vs Gemox	0.595 (0.312–1.135)	0.099	0.547 (0.264–1.131)	0.103
**Age**				
>58 vs ≤58	1.089 (0.567–2.092)	0.798		
**Sex**				
male vs female	0.896 (0.650–1.235)	0.504		
**ECOG**				
1 vs 0	1.709 (0.883–3.310)	0.112	2.148 (1.028–4.488)	**0.042**
**Primary tumor sites**				
Intrahepatic	Ref			
Hilar	1.609 (0.827–3.129)	0.161	2.123 (1.023–4.402)	**0.043**
Distal	0.893 (0.207–3.846)	0.879	0.890 (0.199–3.983)	0.878
**Disease status**				
Metastasis vs Locally advanced	0.939 (0.660–1.335)	0.724		
**Liver metastasis**	1.393 (0.716–2.710)	0.329		
**CA19–9**				
>40 vs ≤40	3.198 (1.237–8.266)	**0.016**	4.972 (1.768–13.980)	**0.002**

Bold values are statistically significant (p ≤ 0.05)

*Ref* reference, *HR* hazard ratio, *CI* confidence interval, *ECOG* Eastern Cooperative Oncology Group

### Safety

The Grade 3–4 treatment-related adverse events were shown in Table 5. Significantly more grade 3–4 vomiting occurred in the mFOLFIRINOX group (mFOLFIRINOX vs Gemox: 7 (25.9%) vs 1 (4.5%), *p* = 0.044). However, grades 3–4 of febrile neutropenia, diarrhea and fatigue occurred only in the mFOLFIRINOX group, while grade 3–4 of thrombocytopenia occurred only in the Gemox group. Treatment was delayed because of toxicity in 11 (40.7%) patients in the mFOLFIRINOX regimen and 3 (13.6%) patients in the Gemox regimen.

**Table 5:** Grade 3–4 Adverse events occurring in patients

Adverse event	mFOLFIRINOX (***n*** = 27) N (%)	Gemox (***n*** = 22) N (%)	***p*** value
Neutropenia	13 (48.1)	8 (36.4)	0.407
Febrile neutropenia	3 (11.1)	0	0.107
Anemia	1 (3.7)	2 (9.1)	0.434
Thrombocytopenia	0	1 (4.5)	0.263
Vomiting	7 (25.9)	1 (4.5)	**0.044**
Diarrhea	3 (11.1)	0	0.107
Peripheral neuropathy	2 (7.4)	3 (13.6)	0.474
Fatigue	1 (3.7)	0	0.362

Bold values are statistically significant (p ≤ 0.05)

## Discussion

To our knowledge, this retrospective study was the first to directly compare the effectiveness between mFOLFIRINOX and Gemox in patients with locally advanced or metastatic CCA. These results suggested that patients received mFOLFIRINOX showed longer PFS than those received with Gemox as a first-line chemotherapy.

Chemotherapy is the preferred choice for locally advanced or metastatic CCA. Commonly used drugs include gemcitabine, fluorouracil and platinum [13]. The low incidence and poor prognosis of CCA resulted in few clinical trials being conducted to compare different chemotherapy regimens, and these studies often included all subgroups of biliary tract cancer. The ABC-02 [4] in 2010 established gemcitabine plus platinum as the first-line systemic therapy for biliary tract cancers. However, the median OS in that study was only 11.7 months, and up-to-now there was no standard second-line treatment. Several studies on chemotherapeutic regimens based on gemcitabine or platinum also failed to further improve survival. In a phase II trial (NCT01375972) [14], the PFS and OS of GEM/S-1 were 5.7 and 10.1 months, respectively. In another phase II study (NCT02527824) [15] using irinotecan, oxaliplatin and S-1 for patients with locally advanced or metastatic biliary tract cancer, the PFS and OS were 6.8 months and 12.5 months, respectively. In another phase II clinical trial in 2018 (NCT02181634) [16], Nab-paclitaxel plus gemcitabine neither improved PFS nor OS (7.7 and 12.4 months). The effectiveness of the Gemox regimen was similar to the gemcitabine-based therapy (PFS: 6.4 months; OS: 12.0 months) in our study.

Previous studies showed that when compared with gemcitabine, FOLFIRINOX prolonged survival for patients with metastatic pancreatic cancer [9, 17]. For advanced biliary tract cancer, a retrospective study showed median OS of patients with first-line FOLFIRINOX was as long as 15 months [11]. In addition, several studies have demonstrated the efficacy and safety of FOLFIRINOX or mFOLFOX as a second-line treatment for advanced CCA [12, 18, 19]. In our study, even when mFOLFIRINOX failed to improve OS compared with Gemox (mFOLFIRINOX vs Gemox, 15.7 vs 12.0 months, *p* = 0.099), PFS in the mFOLFIRINOX regimen was significantly prolonged compared with the Gemox regimen (mFOLFIRINOX vs Gemox, 9.9 vs 6.4 months, *p* = 0.040). Moreover, the mFOLFRINOX regimen resulted in higher ORR (33.3% vs 22.7%) and DCR (77.8% vs 63.5%) than the Gemox regimen. These results suggested better treatment effectiveness using mFOLFIRINOX in patients with locally advanced or metastatic CCA compared to first-line Gemox regimen.

The role of CA19–9 as a prognostic factor for CCA is controversial. In a retrospective study on 344 patients with intrahepatic CCA, CA19–9 has been found to be an independent predictive factor and it was subsequently incorporated into a prognostic score [20]. In another retrospective study on 2816 patients, elevated CA19–9 was found to be an independent risk factor for mortality in intrahepatic CCA [21]. Nevertheless, CA19–9 has also been found to be elevated in non-malignant biliary tract diseases, such as obstructive jaundice or cholangitis [22, 23]. In this study, liver metastases and a high level of CA19–9 were determined to be independent poor prognostic factors.

mFOLFIRINOX is a four-drug regimen that has raised concerns about its adverse drug reactions. In this study, only the incidence of vomiting was increased in the mFOLFIRINOX group. More treatment delays were required in the mFOLFIRINOX group. Irinotecan and high doses of fluorouracil were the likely causes for vomiting. These results are consistent with the studies reported on pancreatic cancer [9, 17, 24, 25]. In general, the toxicity of mFOLFIRINOX was tolerable.

Treatment for advanced CCA remains challengeable, and palliative therapy is the main treatment option. This study shows that mFOLFIRINOX results in promising outcomes for efficacy and safety among patients with advanced CCA. Additionally, this study could constitute the groundwork for establishing more effective sequential systemic chemotherapeutic regimen. Notwithstanding, we need to better understand which agents and which combinations of drugs are most effective and best tolerated. Targeted therapy and immunotherapy are promising therapy but needs further research.

This study has limitations. This is a retrospective study based on a variety of patient population and a small number of patients in a single hospital, which has the inherent defects of proneness to selection biases and possibilities of introducing confounding factors. Nonrandomized analysis makes the results prone to confounding and selection bias. Due to incomplete identification of adverse events, the safety profiles may have missing/incorrect data. The patient’s choice of treatment and the doctor’s recommendation might bias the outcome. As a result of these limitations, our findings must be interpreted with caution. A phase II/III study (PRODIGE 38) [10] comparing mFOLFIRINOX with gemcitabine plus cis-platinum for locally advanced or metastatic biliary tract cancer is ongoing and may provide more convincing evidence.

In conclusion, the prognosis of patients with locally advanced or metastatic CCA was poor, first-line mFOLFIRINOX offered more promising results compared to Gemox. Further prospective evaluation might provide more compelling results.

## Data Availability

The data used in the current study are available from the corresponding author (laoniulyb@shsmu.edu.cn) on reasonable request.

## Abbreviations

CCA: Cholangiocarcinoma
mFOLFIRINOX: Modified FOLFIRINOX
Gemox: Gemcitabine plus oxaliplatin
ECOG: Eastern Cooperative Oncology Group
DCR: Disease control rate
PFS: Progression free survival
OS: Overall survival
CI: Confidence interval
HR: Hazard ratio
CA19–9: Carbohydrate antigen 19–9
